# Supplementary material for: Versatile Capillary Cells for Handling Concentrated Samples in Analytical Ultracentrifugation
Source: Anal Chem. 2024 Feb 1;96(6):2567–73. doi: 10.1021/acs.analchem.3c05006 (PMC10867799; doi:10.1021/acs.analchem.3c05006)
Supplement: Supplementary file 1 — ac3c05006_si_001.pdf [file ac3c05006_si_001.pdf]

Supporting Information

VERSATILE CAPILLARY CELLS FOR HANDLING CONCENTRATED SAMPLES IN ANALYTICAL ULTRACENTRIFUGATION

QUY ONG, XUFENG XU and FRANCESCO STELLACCI*

Laboratory of Supramolecular Nanomaterials and Interfaces, Ecole Polytechnique Fédérale de Lausanne (EPFL), Station 12, 1015 Lausanne, Switzerland.

*Corresponding author

Quy Ong

Laboratory of Supramolecular Nanomaterials and Interfaces, Ecole Polytechnique Fédérale de Lausanne (EPFL), Station 12, 1015 Lausanne, Switzerland.

Email: quy.ong@epfl.ch

Telephone: +4121631002

Table of Content

1. Pictures of the short and long channel designs S3

2. Variation of optical path length S4

3. Comparison of the plots of equation of state S5

4. Cell-stand design and dimensions for working with capillary cells S6

5. Dimensions of the capillary cell by laser etching S7

6. Dimensions of the capillary cell by mechanical drilling S8

7. Dimensions of a spacer to adjust the centerpiece height S9

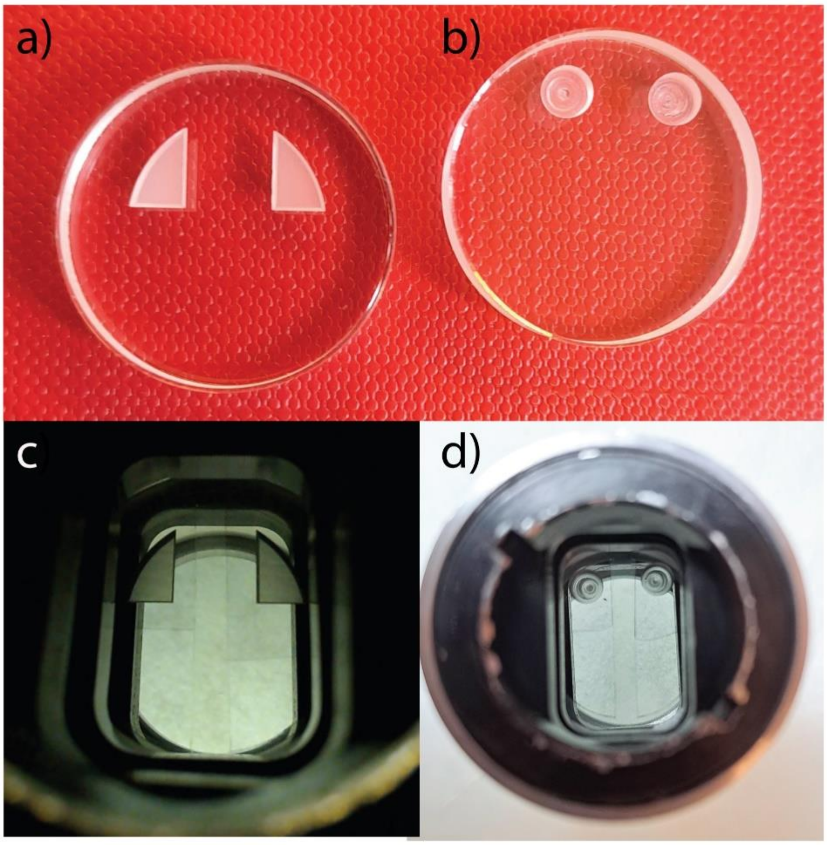

Figure S1. Pictures of the short and long channel designs. Variation of channel length is based on the amount of liquids and window design. In short-column design (a) from (c) one can see the two menisci showing different channel lengths from the reference channel (3µl buffer used) and sample channel (2.5µl sample used), respectively. Here the reservoirs were made from laser etching. Mechanical drilling of cylindrical wells into the sapphire windows could also be used to make the reservoirs shown in (b). (d) shows the complete liquid channels where 6µl and 5.5 µl of buffer and sample are used, respectively.

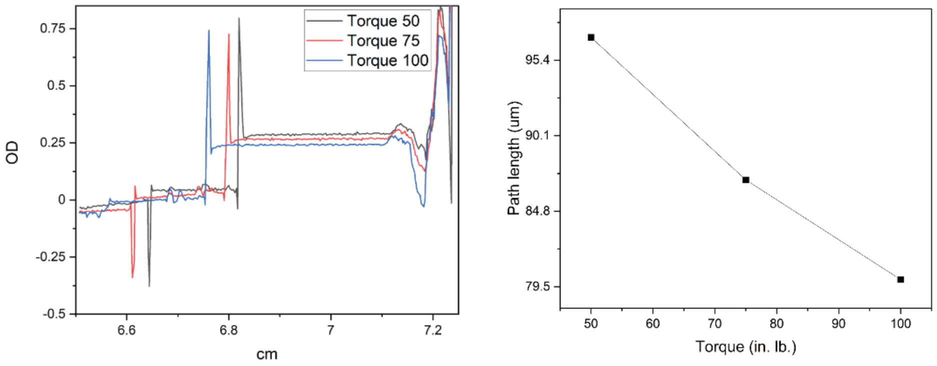

Figure S2. Variation of optical path length. Torque 50, 75, or 100 in. lb resulted in the cell thickness of 97 µm, 87 µm, or 80 µm, respectively. The sample used was BSA 43 mg/ml in PBS 1x.

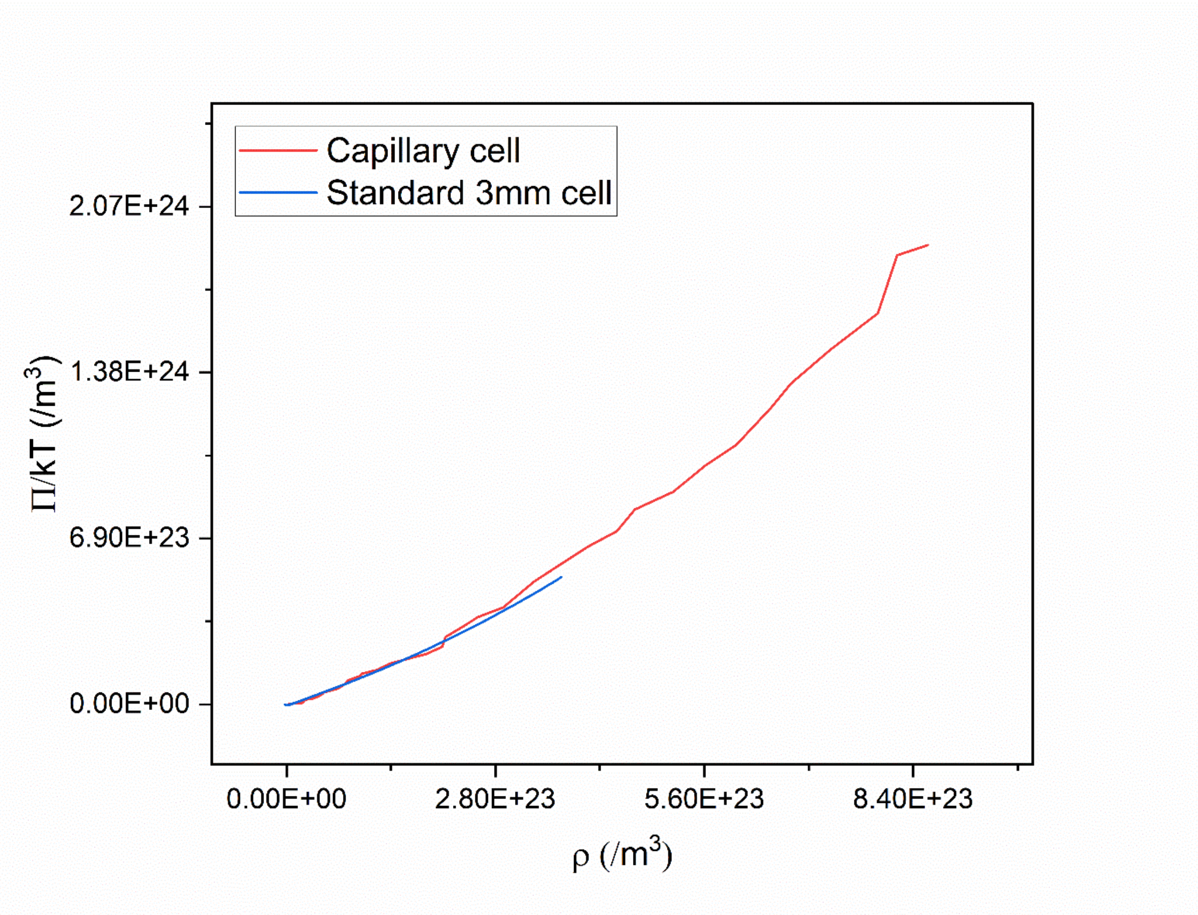

Figure S3. Comparison of the plots of equation of state for BSA obtained from a 3 mm-centerpiece cell and a capillary-centerpiece cell. The capillary cell made accessible higher particle density.

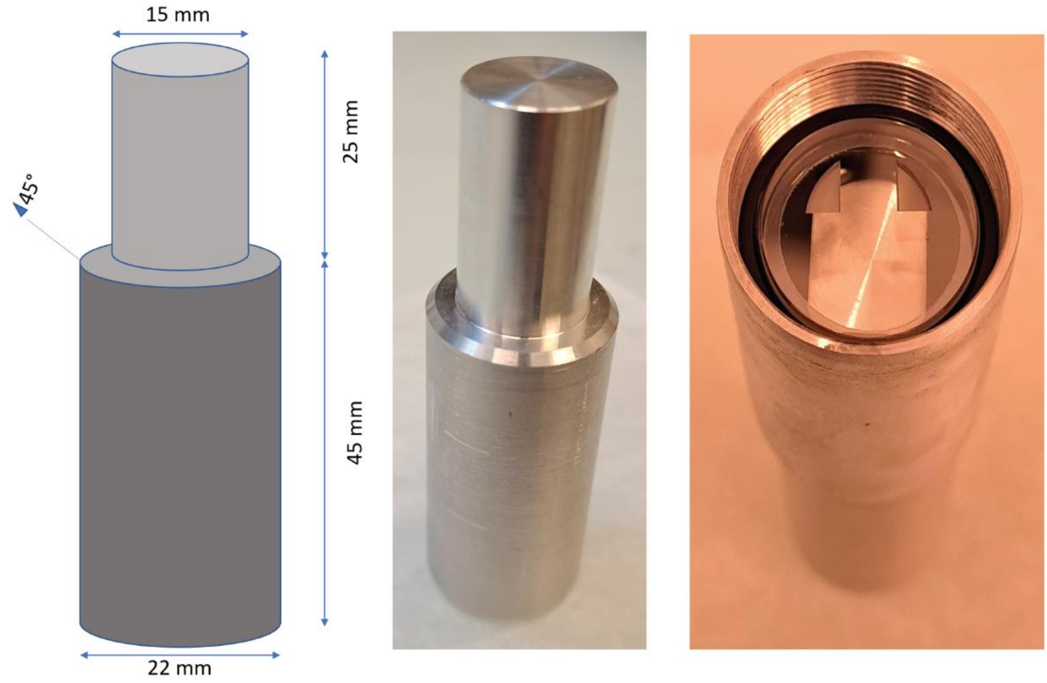

Figure S4. Cell-stand design and dimensions for working with capillary cells. Here the bottom window partially slides into the gliding rail.

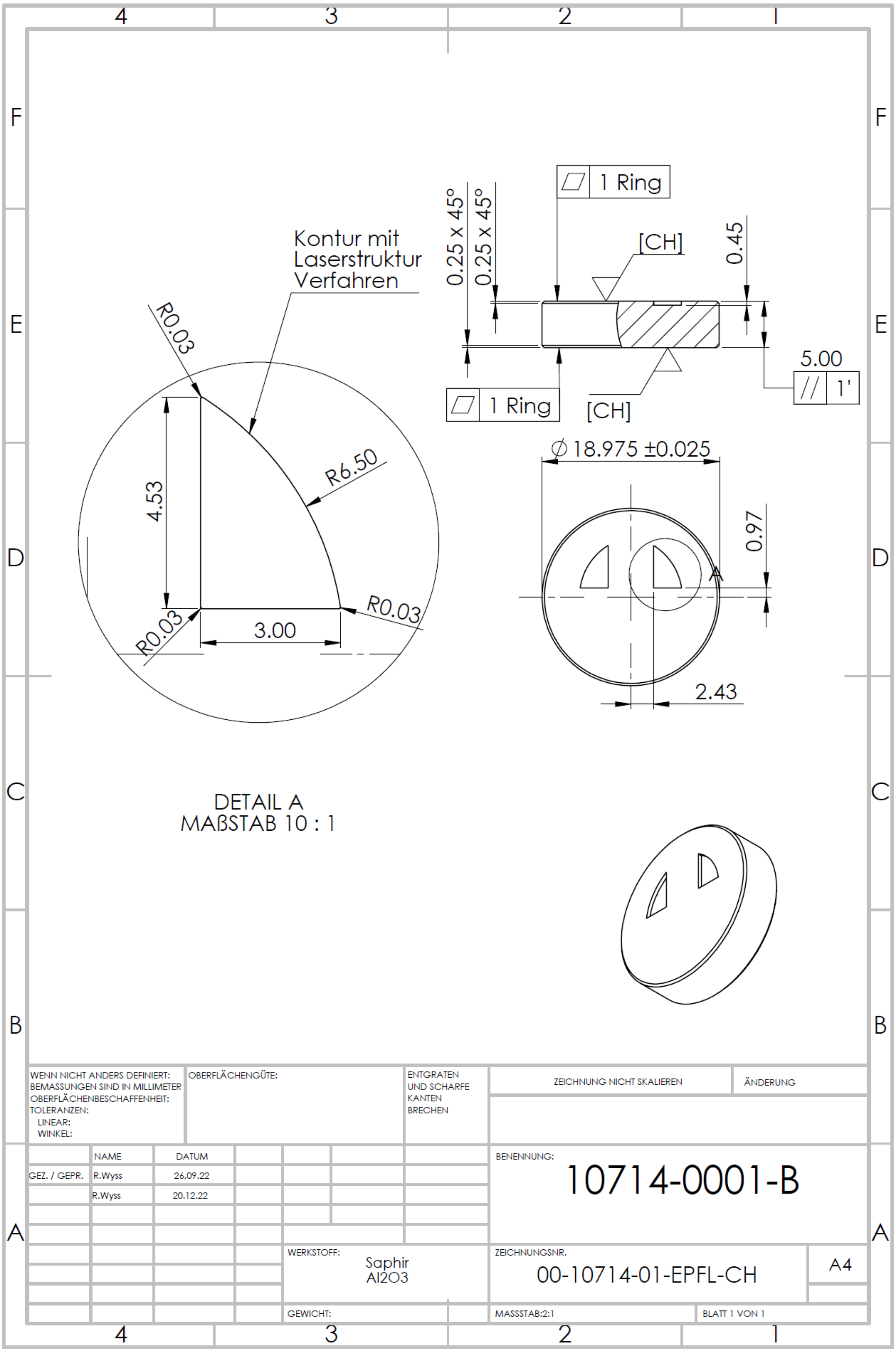

Figure S5. Dimensions of the capillary cell by laser etching presented in this study.

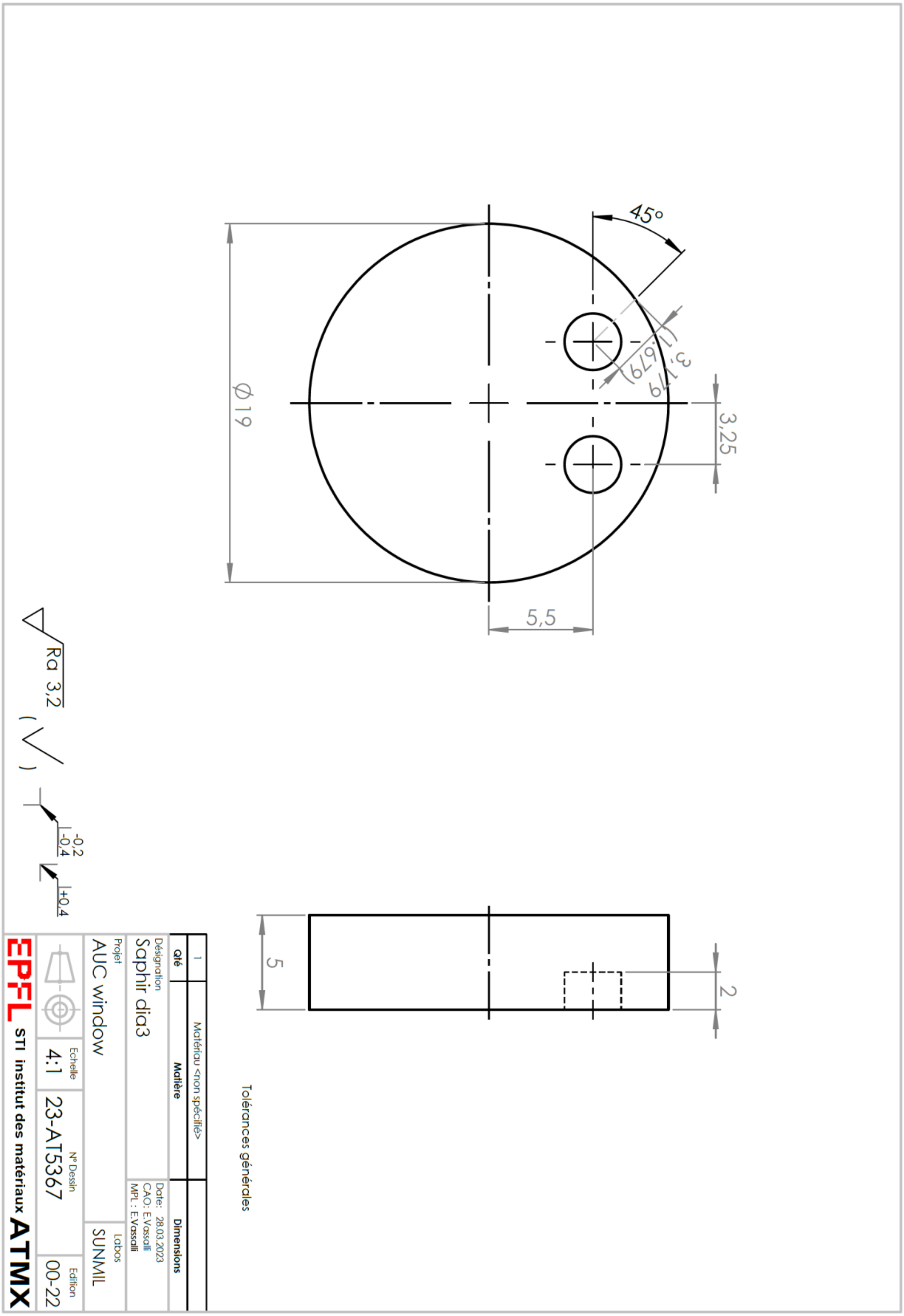

Figure S6. Dimensions of the capillary cell by mechanical drilling presented in this study.

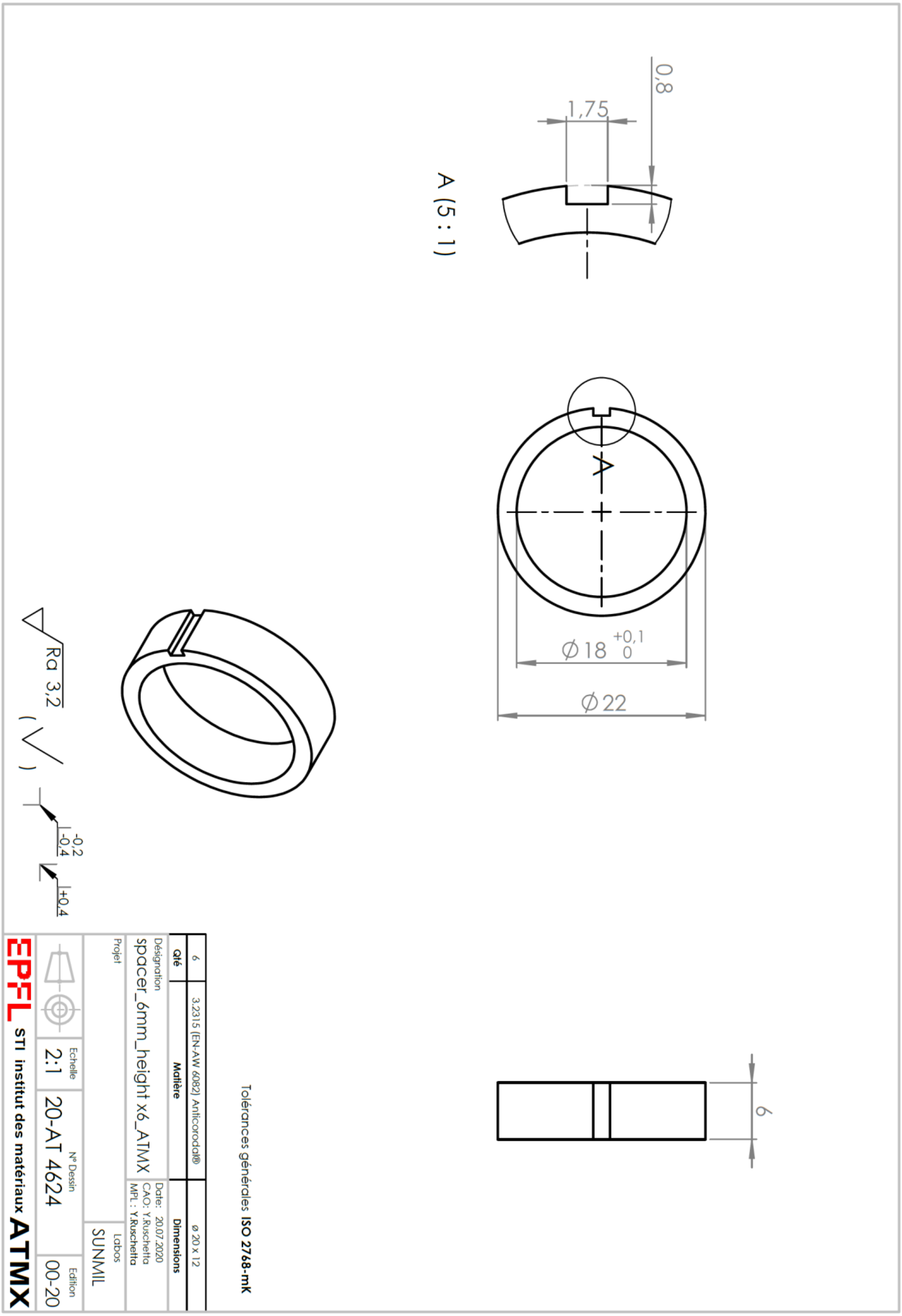

Figure S7. Dimensions of a spacer to adjust the centerpiece height.
